# Perceptions of Treatment Burden Among Elders With Diabetes and Comorbid Alzheimer’s Disease and Related Dementias

**DOI:** 10.1093/geroni/igaa057.882

**Published:** 2020-12-16

**Authors:** Victoria Vaughan Dickson, Halia Melnyk, Rosie Ferris, Joshua Chodosh, Caroline Blaum

**Affiliations:** 1 New York University, New York, New York, United States; 2 Nursing, New York, New York, United States; 3 NYU Langone Health, New York, New York, United States; 4 New York University Grossman School of Medicine, New York, New York, United States

## Abstract

Background: An estimated 25% of older adults with diabetes (DM) may have co-occurring Alzheimer’s Disease and Related Dementias (ADRD), complicated by multiple treatment plans and providers. Assessing treatment burden has been limited to patients’ perspectives; little is known about caregiver perceptions of treatment burden despite their important role in personal care and treatment adherence. The purpose of this qualitative study was to describe caregiver perceptions of treatment burden for older adults with DM-ADRD. Methods: This qualitative study was conducted in the formative phase of “Enhanced Quality in Primary care for Elders with DM-ADRD (EQUIPED-ADRD) a pragmatic randomized controlled trial in a large, diverse healthcare system. A diverse sample of caregivers (n=15) of patients enrolled in the RCT participated in interviews about their caregiver role and perceptions of treatment burden of DM-ADRD clinical management. Qualitative data were analyzed using content analysis and themes about treatment burden were compared to domains on the Treatment Burden Questionnaire (TBQ). Results: Caregivers reported high levels of burden related to treatment plans for patients with DM-ADRD. Themes related to complexity and burden of medication management, monitoring (e.g., blood pressure, glucose monitoring), dietary and physical activity regimens, navigating healthcare providers and financial burden were reported. Caregivers also described high levels of emotional burden that was associated with patient’s cognitive decline and family functioning stress. Conclusions: Interventions to reduce treatment burden for patients and caregiver should include activating social/nursing services, respite care and care coordination that may support caregivers especially as patient treatment increases in complexity over time.

